# Sex-Specific Effects of Vitamin D Status on the Metabolic Profile in Prediabetic Subjects

**DOI:** 10.1155/2021/2811756

**Published:** 2021-10-05

**Authors:** Teresa Gisinger, Michael Leutner, Evelyne Wohlschläger-Krenn, Robert Winker, Sonja Nistler, Georg Endler, Alexandra Kautzky-Willer

**Affiliations:** ^1^Unit of Gender Medicine, Clinical Division of Endocrinology and Metabolism, Department of Medicine III, Medical University of Vienna, Waehringer Guertel 18-20, 1090 Vienna, Austria; ^2^Health and Prevention Center KFA, Vienna, Austria; ^3^Gruppenpraxis Labors.at, Vienna, Austria; ^4^Gender Institute, 3571 Gars am Kamp, Austria

## Abstract

**Introduction:**

We aim to investigate the effect of vitamin D on metabolic parameters in a population with prediabetes and to detect possible sex differences.

**Methods:**

In 621 patients with diagnosed prediabetes, glucose, lipid, and anthropometric parameters were measured. Furthermore, the interaction of 25-OH-vitamin D (25-hydroxyvitamin D) with metabolic and glucose metabolism parameters was analysed in the total prediabetic population, as well as after stratification by sex (female vs. male prediabetic subgroup), by logistic regression.

**Results:**

25-OH-vitamin D was negatively related to cholesterol, BMI, fatty liver index, insulin, and HOMA-IR. Especially in the male prediabetic cohort, 25-OH-vitamin D levels negatively correlated with total cholesterol levels (*r* = −0.17, *p*=0.001), with triglycerides (*r* = −0.17, *p*=0.001), and with HbA1c levels (*r* = −0.14, *p*=0.010). Only in the female cohort with prediabetes, we found a negative correlation of 25-OH-vitamin D levels with systolic (*r* = −0.18, *p*=0.005) and diastolic blood pressures (*r* = −0.23, *p* < 0.001).

**Conclusion:**

In this study, in females with prediabetes, 25-OH-vitamin D was notably related to a more favourable metabolic profile, including lower total cholesterol and higher HDL cholesterol levels. On the contrary, in men with prediabetes, there was a stronger association between 25-OH-vitamin D and cholesterol-HDL quotient, as well as fatty liver index was observed in the male prediabetic subgroup. Therefore, sex differences should be considered in future studies on vitamin D and glucose tolerance status.

## 1. Introduction

Vitamin D is an important micronutrient for human health. Still, around 13% of the European population suffer from vitamin D deficiency [1]. Nowadays, various studies investigated the necessity of vitamin D for numerous nonskeletal effects, including the insulin secretion in the pancreas [2]. The vitamin D receptor (VDR) is present in the pancreatic *β*-cells and in tissue influenced by insulin as the skeletal muscle, myocardium, and adipose tissue [3, 4]. Altered insulin secretion and sensitivity may be associated with polymorphisms of VDR [5]. Vitamin D might also have a protective role against pancreatic *β*-cell inflammatory damage and death by immunomodulatory properties [6]. There is also evidence that low levels of serum 25-hydroxyvitamin D (25-OH-vitamin D), an accepted indicator of vitamin D status, are associated with impaired glucose tolerance and diabetes mellitus [7]. Furthermore, studies claim that vitamin D status is associated with diabetic complications including nephropathy, retinopathy, and neuropathy [8].

In 2017, the National Diabetes Statistics Report from the US Centers for Disease Control and Prevention estimates that approximately 34% of the US adults suffer from prediabetes [9]. Prediabetes is the prestage state for developing diabetes mellitus [10]. A Chinese study described that participants with low levels of 25-OH-vitamin D have a higher risk of developing diabetes or prediabetes [11]. Furthermore, Gao et al. evidenced low levels of 25-OH-vitamin D already 4 years prior to the diagnosis of prediabetes or diabetes mellitus [11]. Also, a Swedish study observed that low levels of 25-OH-vitamin D are associated with higher incident diabetes mellitus in men and women with prediabetes [12]. However, after adjusting for all possible confounders, this effect was only significant in men, possibly based on differences in BMI [12]. Furthermore, a Chinese study reported that vitamin D has a negative impact on insulin resistance, but this could only be shown in male patients with newly diagnosed type 2 diabetes mellitus [13]. In the female subgroup and in the general population, no significant effect could be shown [13]. A prior study analysed that daily vitamin D supplementation in participants with prediabetes could reduce the risk for developing overt diabetes [14].

Nevertheless, the current knowledge on the impact of vitamin D on prediabetes is scarce, especially concerning the association with metabolic profile and possible sex-specific differences. Hence, the aim of the present study was to investigate the effect of vitamin D levels on the outcome of prediabetes and to explore sex-specific effects.

## 2. Subject, Materials, and Methods

### 2.1. Study Design

The present cross-sectional study is a retrospective data analysis of first visits to the prediabetes clinic at “Sanatorium Hera” in Vienna from October 2015 to September 2017. The patients underwent a standard medical checkup with physical examination, electrocardiogram, extensive blood analysis, and assessment of vital parameters after giving informed consent. Only patients with prediabetes were included (*n* = 621). In order to classify prediabetes, a glucose tolerance test (oGTT) was carried out, or fasting blood glucose levels and HbA1c values were used for the diagnosis. Prediabetes was defined according to the guidelines of the American Diabetes Association [15] if HbA1c was ≥5.7% and <6.5% and/or if they had fasting blood glucose levels ≥100 mg/dl and <126 mg/dl and/or ≥140 mg/dl and <200 mg/dl in a 2-hour oral glucose tolerance test.

### 2.2. Calculations

The homeostasis model assessment insulin resistance score (HOMA-IR) was calculated by using basal insulin and glucose levels (HOMA-IR = Ins *x* Gluc/405). The fatty liver index (FLI) was calculated by body mass index (BMI), waist circumference, *γ*-GT, and triglycerides (FLI = (e ^0.953^^*∗*^^loge (triglycerides) + 0.139^^*∗*^^BMI + 0.718^^*∗*^^loge (ggt) + 0.053^^*∗*^^waist circumference - 15.745^)/(1 + e ^0.953^^*∗*^^loge (triglycerides) + 0.139^^*∗*^^BMI + 0.718^^*∗*^^loge (ggt) + 0.053^^*∗*^^waist circumference - 15.745^) ^*∗*^ 100) [16].

### 2.3. Statistical Analysis

At first, the data dictionary was scanned for variables suitable for analysis. The variables were defined as metabolic parameters and parameters relevant for glucose metabolism. In general, in the prediabetes cohort, a total of 166 participants had a low 25-OH-vitamin D level defined as < 20 ng/ml, and 40 participants had a vitamin D deficiency defined as <12 ng/ml. Next, the population was divided by 25-OH-vitamin D status. Two cohorts were defined. First, “the lower 25-OH-vitamin D cohort” (*n* = 308) is defined as 25-OH-vitamin D < 26.5 ng/ml. Second, “the higher 25-OH-vitamin D cohort” (*n* = 309) is defined as 25-OH-vitamin D ≥ 26.5 ng/ml. The cohorts were split according to the median of 25-OH-vitamin D status (median = 26.5 ng/ml). The range of 25-OH-vitamin D was 6.7. Frequency, mean, and standard deviation were calculated for every parameter. Furthermore, interaction terms between 25-OH-vitamin D and all parameters were explored to determine if the impacts of the variables differed for lower and higher 25-OH-vitamin D groups by a linear and logistic regression model. Then, sex-stratified analysis was performed. The male cohort consists of 375 individuals, and the female cohort consists of 246 individuals. Mean and standard deviation were calculated for every parameter. As the next step, the interaction of 25-OH-vitamin D and sex with all parameters was analysed by linear regression. Lastly, the interaction of only 25-OH-vitamin D with all parameters was explored. For all analyses, statistical significance was defined with a *p* value <0.05. Analyses were carried out by SPSS Statistics version 26.

## 3. Results

In total, 621 individuals were included in the present study. In Table 1, 25-OH-vitamin D status and metabolic parameters are presented. The general population was grouped into a lower and a higher 25-OH-vitamin D cohort. In comparison to the lower 25-OH-vitamin D cohort, lower means of total cholesterol (205.67 vs. 214.03 mg/dl), LDL cholesterol (122.45 vs. 132.25 mg/dl), triglyceride levels (105.3 vs. 124.35 mg/dl), cholesterol-HDL quotient (3.46 vs. 4.03 mg/dl), higher systolic and diastolic blood pressures (138.96 mmHg vs. 135.91 mmHg and 85.24 mmHg vs. 82.72 mmHg), and BMI (27.44 kg/m^2^ vs. 29.15 kg/m^2^) were seen in the higher 25-OH-vitamin D subgroup. Furthermore, the higher 25-OH-vitamin D cohort featured higher means of HDL cholesterol (62.68 vs. 56.65 mg/dl).

Table 2 shows sex-specific data of the prediabetic population. In the female compared to the male cohort, a higher mean concentration of 25-OH-vitamin D (29 vs. 26.96 ng/ml), total cholesterol (216.56 vs. 206.19 mg/dl), LDL cholesterol (129.56 vs. 126.95 mg/dl), and HDL cholesterol (64.81 vs. 55.90 mg/dl) could be observed. Nevertheless, the female cohort had lower mean abdominal circumference (99.85 vs. 102 cm), fatty liver index (50.65 vs. 59.03), and glucose levels (105.25 vs. 107.44 mg/dl). In addition, the male and female cohorts were analysed in respect of 25-OH-vitamin D levels. 25-OH-vitamin D had a negative effect on cholesterol (*r* = −0,18, *p* < 0.001), especially in women. 25-OH-vitamin D had a negative effect on cholesterol-HDL quotient (*r* = −0.3, *p* < 0.001) and fatty liver index (*r* = −0.27, *p* < 0.001), particularly in men.

Table 3 reports the correlation of 25-OH-vitamin D with the metabolic parameters by logistic regression models. In the general cohort, a negative correlation was evidenced between the 25-OH-vitamin D level and triglycerides (*r* = −0.14, *p* < 0.001), HDL cholesterol (*r* = 0.23, *p* < 0.001), LDL cholesterol (*r* = −0.17, *p* < 0.001), cholesterol-HDL quotient (*r* = −0.26, *p* < 0.001), total cholesterol (*r* = −0.11, *p*=0.009), BMI (*r* = −0.18, *p* < 0.001), abdominal circumference (*r* = −0.18, *p* < 0.001), fatty liver index (*r* = −0.19, *p* < 0.001), insulin levels (*r* = −0.19, *p* < 0.001), and HOMA-IR (*r* = −0.19, *p*=0.001).

**Figure 1:**
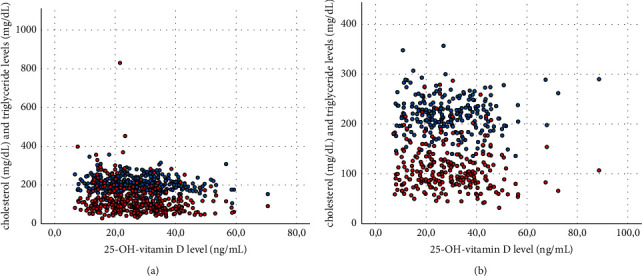
Scatter plots of vitamin D with cholesterol (blue) and triglyceride (red) levels in the male (a) and female (b) cohort.

**Figure 2:**
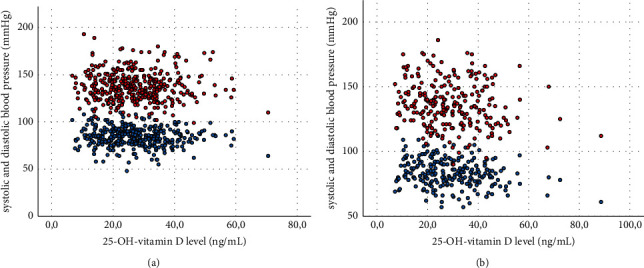
Scatter plots of vitamin D with systolic (red) and diastolic (blue) blood pressures in the male (a) and female (b) cohort.

In the sex-segregated analysis, 25-OH-vitamin D was also negatively related to triglycerides (*r* = −0.17, *p*=0.001, Figures 1(a) and 1(b)), HDL cholesterol (*r* = 0.16, *p*=0.002), LDL cholesterol (*r* = −0.18, *p* < 0.001), cholesterol-HDL quotient (*r* = −0.24, *p* < 0.001), total cholesterol (*r* = −0.17, *p*=0.001, Figures 1(a) and 1(b)), BMI (*r* = −0.17, *p*=0.001), abdominal circumference (*r* = −0.16, *p*=0.002), fatty liver index (*r* = −0.16, *p*=0.007), insulin (*r* = −0.19, *p*=0.001), and HOMA-IR (*r* = −0.18, *p*=0.012) in the male cohort alone. Additionally, in the male cohort, a negative correlation of 25-OH-vitamin D with glucose (*r* = 0.99, *p*=0.001) and HbA1c levels (*r* = −0.14, *p*=0.010) was found. In the female cohort only, a negative correlation of 25-OH-vitamin D with HDL cholesterol (*r* = 0.27, *p* < 0.001), LDL cholesterol (*r* = −0.15, *p*=0.017), cholesterol-HDL quotient (*r* = −0.28, *p* < 0.001), BMI (*r* = −0.24, *p* < 0.001), abdominal circumference (*r* = −0.19, *p*=0.003), fatty liver index (*r* = −0.21, *p*=0.005), insulin levels (*r* = −0.17, *p*=0.026), HOMA-IR (*r* = −0.20, *p*=0.022), systolic blood pressure (*r* = −0.18, *p*=0.005, Figures 2(a) and 2(b)), and diastolic blood pressure (*r* = −0.23, *p* < 0.001, Figures 2(a) and 2(b)) was evaluated.

## 4. Discussion

To summarize, our study confirmed that higher 25-OH-vitamin D levels are related to a more favourable metabolic profile in patients with prediabetes. 25-OH-vitamin D was negatively related to total cholesterol, LDL cholesterol, triglyceride levels, cholesterol-HDL quotient, BMI, abdominal circumference, fatty liver index, blood pressure values, insulin levels, and HOMA-IR. Furthermore, we observed sex differences regarding the relationship of 25-OH-vitamin D with total cholesterol, triglycerides, HbA1c levels, and systolic and diastolic blood pressures.

Bearing in mind that our study population consists of subjects with prediabetes, the effect of 25-OH-vitamin D on glucose metabolism is of importance, expanding the knowledge on possible vitamin D effects to a group at a high risk of progression to diabetes. More specifically, we could show that higher levels of 25-OH-vitamin D were related to a lower HOMA-IR and lower levels of insulin and HbA1c in the whole study population. Further analysing sex-specific differences, we could observe higher HbA1c values in the female population and a negative relationship of 25-OH-vitamin D with HbA1c values in the female prediabetes cohort. Previous studies support our findings [17–19]. These studies investigated the general population or patients with overt diabetes mellitus. The aforementioned studies claimed that vitamin D may decrease insulin resistance, fasting glucose, insulin levels, and HOMA-IR [17–19]. However, in the general population, no effect of vitamin D on HbA1c levels could be found [19]. Vitamin D's effect on insulin, HbA1c, and HOMA-IR can be explained by the ability of 25-OH-vitamin D to bind on pancreatic *β*-cells and therefore alter their function in a positive way [2]. A prior study showed that daily vitamin D supplementation in participants with prediabetes could reduce their risk of developing overt diabetes [14]. A new finding of our study is that we studied sex differences in the association of 25-OH-vitamin D status and metabolic parameters in a cohort with prediabetes.

Various studies analysed the effect of vitamin D on metabolic parameters. Vitamin D in combination with probiotics increased HDL cholesterol levels in patients with diabetes mellitus [17]. Other studies reported that vitamin D alone may increase HDL cholesterol levels in hypertensive patients [20]. In our study, we found similar results in a population with prediabetes, especially in women. Concerning total cholesterol and LDL cholesterol, previous studies had divergent results. An earlier study reported higher levels of total cholesterol and LDL cholesterol related to higher vitamin D levels in a population diagnosed with arterial hypertension [20]. On the contrary, lower levels of total cholesterol and LDL cholesterol were described following vitamin D supplementation in an older Lebanese population [19]. In our study, 25-OH-vitamin D was negatively related to total cholesterol and LDL cholesterol levels in a population with prediabetes. Particularly in the female population, we could see a decrease in total cholesterol with higher 25-OH-vitamin D. Vitamin D might be able to alter the gene expression of apolipoproteins [21] and thus affect cholesterol levels. Previous studies investigated that higher cholesterol levels are associated with higher fat mass. Notably in women, we confirmed such an association potentially because women generally have a higher percentage of subcutaneous fat [22]. Further previous studies investigated a positive correlation of fat mass on LDL and HDL cholesterol levels [23, 24]. Moreover, former studies provided knowledge that vitamin D can inversely affect obesity and BMI [18]. In our study, lower BMI values were also related to higher 25-OH-vitamin D levels. The reason why vitamin D is lower in individuals with higher BMI levels could be that it is stored in the excess fat mass of obese subjects [25]. Another hypothesis is that individuals with higher BMI values might have a higher distribution volume and therefore require greater dietary intake of vitamin D as compared to lean individuals [26]. Previously, it could be observed that vitamin D intake lowers the risk for nonalcoholic fatty liver disease [27]. In addition, men have a higher risk of fatty liver disease as oestrogen might exert protective effects [28]. Therefore, it is not surprising that we observed a negative association of 25-OH-vitamin D on the fatty liver index, particularly in men.

In the present study, we have to report some limitations. First, we lack a control group without prediabetes. Secondly, we only measure vitamin D levels at one time point in a cross-sectional design. Thirdly, the database did not ask for menopausal status. Strength of the present study is the rather large number of subjects with documented prediabetes and that, to the best of our knowledge, it is the first study investigating the association of vitamin D with a variety of metabolic parameters and glucose metabolism in this specific population, including analysis of potential sex differences. Various studies investigated vitamin D levels and their impact on patients with diabetes mellitus. A previous study could show that vitamin D supplementation in a population with prediabetes does not lead to a lower risk of diabetes mellitus [14]. Still, the findings of our study could lead to greater awareness of sex differences in the association of 25-OH-vitamin D levels with metabolic parameters in a cohort of patients with prediabetes. The results may emphasize the importance of vitamin D measurements and possibly supplementation in case of deficiency in prediabetic patients. In the current COVID-19 pandemic, patients with impaired glucose metabolism are even a more important target group of research as patients with diabetes mellitus not only have a higher incidence of COVID-19 but also a higher mortality rate, especially males [29]. Furthermore, studies suggested that vitamin D supplementation might be able to reduce risk for COVID-19 and progression to severe disease and mortality [30]. Therefore, the present study may contribute to further understand the impact of vitamin D on metabolism in this vulnerable population. Additionally, our sex-specific results of vitamin D's association with metabolism could help to improve gender-sensitive care for the population with prediabetes.

## Figures and Tables

**Table 1:** Frequency of sex and mean and standard deviation of all parameters in a cohort with lower 25-OH-vitamin D levels and higher 25-OH-vitamin D levels.

Variable	Lower vitamin D (*n* = 308)	Higher vitamin D (*n* = 313)	*p* value
Age	56.02 (±11.24)	60.87 (±9.55)	<0.001
BMI (kg/m^2^)	29.16 (±4.8)	27.53 (±4.27)	<0.001
Waist circumference (cm)	102.77 (±11.60)	99.60 (±11.71)	<0.001
Sex			
Male	192 (62.3%)	183 (58.5%)	0.033
Female	116	130	
25-Hydroxy-vitamin D (ng/ml)	18.54 (±5.17)	36.84 (±8.67)	—
Cholesterol (mg/dl)	214.24 (±38.98)	206.37 (±38.83)	0.009
HDL cholesterol (mg/dl)	56.68 (±15.85)	62.11 (±15.91)	<0.001
LDL cholesterol (mg/dl)	132.25 (±35.22)	123.78 (±34.55)	<0.001
Triglycerides (mg/dl)	125.20 (±75.02)	104.63 (±46.08)	<0.001
Cholesterol-HDL quotient	4.03 (±1.32)	3.50 (±0.98)	<0.001
HbA1c (%)	5.57 (±0.36)	5.54 (±0.33)	0.062
Glucose (mg/dl)	106.40 (±9.46)	106.76 (±8.10)	0.539
Glucose tolerance test (fasted)	108.09 (±9.04)	108.00 (±7.92)	0.339
Glucose tolerance test (2 h)	118.28 (±31.46)	120.80 (±31.97)	0.880
Insulin (uU/ml)	14.06 (±8.99)	11.46 (±6.13)	<0.001
Fatty liver index	59.79 (±26.43)	50.58 (±26.61)	<0.001
HOMA-IR	3.85 (±2.48)	3.12 (±1.81)	0.001
Systolic blood pressure (mmHg)	138.96 (±15.89)	135.91 (±16.75)	0.007
Diastolic blood pressure (mmHg)	85.24 (±10.71)	82.72 (±9.94)	<0.001

Lower 25-OH-vitamin D cohort is defined by a 25-OH-vitamin D level <26.5 ng/ml. Higher 25-OH-vitamin D cohort is defined by a 25-OH-vitamin D level ≥ 26.5 ng/ml. HDL: high-density lipoprotein; LDL: low-density lipoprotein; BMI: body mass index; HOMA-IR: homeostasis model assessment.

**Table 2:** Mean and standard deviation of all parameters in the male and the female cohort.

Variable	Male (*n* = 375)	Female (*n* = 246)	*p* value
Age	58.17 (±11.12)	58.92 (±10.01)	0.393
BMI (kg/m^2^)	28.07 (±3.9)	28.74 (±5.54)	0.078
Waist circumference (cm)	102 (±10.59)	99.85 (±13.28)	0.028
25-Hydroxy-vitamin D (ng/ml)	26.96 (±10.51)	29 (±13.05)	0.033
Cholesterol (mg/dl)	206.19 (±39.69)	216.56 (±37.3)	0.001
HDL cholesterol (mg/dl)	55.90 (±15.05)	64.81 (±16.18)	<0.001
LDL cholesterol (mg/dl)	126.95 (±35.57)	129.56 (±34.41)	0.368
Triglycerides (mg/dl)	117.91 (±72.28)	110.16 (±44.8)	0.135
Cholesterol-HDL quotient	3.93 (±1.3)	3.52 (±0.96)	<0.001
HbA1c (%)	5.5 (±0.34)	5.64 (±0.33)	<0.001
Glucose (mg/dl)	107.44 (±8.35)	105.25 (±9.35)	0.003
Oral glucose tolerance test (oGTT fasted)	108.53 (±8.2)	107.32 (±9.05)	0.138
Oral glucose tolerance test (oGTT 2 h)	118.36 (±31.05)	120.92 (±32.62)	0.399
Insulin (uU/ml)	12.75 (±8.46)	13.19 (±7.17)	0.571
Fatty liver index	59.03 (±26.25)	50.65 (±27.11)	0.001
HOMA-IR	3.57 (±2.33)	3.45 (±2.08)	0.625
Systolic blood pressure (mmHg)	137.81 (±15.48)	136.80 (±17.74)	0.464
Diastolic blood pressure (mmHg)	84.36 (±10.27)	83.35 (±10.58)	0.246

HDL: high-density lipoprotein; LDL: low-density lipoprotein; BMI: body mass index; HOMA-IR: homeostasis model assessment.

**Table 3:** Correlation of 25-OH-vitamin D on the mentioned variables and all *r* and *p* values of the linear regression on 25-OH-vitamin D and sex on all mentioned parameters.

Correlation of 25-OH-vitamin D	General cohort		Male cohort		Female cohort	
Variable	*r*	*p* value	*r*	*p* value	*r*	*p* value
BMI (kg/m^2^)	−0.20	<0.001	−0.17	0.001	−0.24	<0.001
Waist circumference (cm)	−0.18	<0.001	−0.16	0.002	−0.19	0.003
Cholesterol (mg/dl)	−0.11	0.009	−0.17	0.001	−0.05	0.48
HDL cholesterol (mg/dl)	0.23	<0.001	0.16	0.002	0.27	<0.001
LDL cholesterol (mg/dl)	−0.17	<0.001	−0.18	<0.001	−0.15	0.017
Triglycerides (mg/dl)	−0.14	<0.001	−0.17	0.001	−0.09	0.18
Cholesterol-HDL quotient	−0.26	<0.001	−0.24	<0.001	−0.28	<0.001
HbA1c (%)	−0.76	0.062	−0.14	0.010	−0.05	0.49
Glucose (mg/dl)	0.25	0.539	0.01	0.99	0.07	0.27
Insulin (uU/ml)	−0.18	<0.001	−0.19	0.001	−0.17	0.026
Fatty liver index	−0.19	<0.001	−0.16	0.007	−0.21	0.005
HOMA-IR	−0.19	0.001	−0.18	0.012	−0.20	0.022
Systolic blood pressure (mmHg)	−0.11	0.007	−0.04	0.450	−0.18	0.005
Diastolic blood pressure (mmHg)	−0.16	<0.001	−0.09	0.078	−0.23	<0.001

HDL: high-density lipoprotein; LDL: low-density lipoprotein.

## Data Availability

The data used to support the findings of this study may be released upon application to the authors.
